# Ponatinib induces a sustained deep molecular response in a chronic myeloid leukaemia patient with an early relapse with a T315I mutation following allogeneic hematopoietic stem cell transplantation: a case report

**DOI:** 10.1186/s12885-018-5100-4

**Published:** 2018-12-07

**Authors:** Nuno Cerveira, Rosa Branca Ferreira, Susana Bizarro, Cecília Correia, Lurdes Torres, Susana Lisboa, Joana Vieira, Rui Santos, Fernando Campilho, Carlos Pinho Vaz, Luís Leite, Manuel R. Teixeira, António Campos

**Affiliations:** 1 Department of Genetics, Portuguese Oncology Institute, Porto, Portugal; 2 Department of Bone Marrow Transplantation, Portuguese Oncology Institute, Porto, Portugal; 3 Institute of Biomedical Sciences (ICBAS), University of Porto, Porto, Portugal

**Keywords:** CML, T315I, Relapse, HSCT, Ponatinib

## Abstract

**Background:**

Atypical *BCR-ABL1* transcripts are detected in less than 5% of patients diagnosed with chronic myeloid leukaemia (CML), of which e19a2 is the most frequently observed, with breakpoints in the micro breakpoint cluster region (μ-BCR) and coding for the p230 BCR-ABL1 protein. p230 CML is associated with various clinical presentations and courses with variable responses to first-line imatinib.

**Case presentation:**

Here we report a case of imatinib resistance due to an E255V mutation, followed by early post-transplant relapse with a T315I mutation that achieved a persistent negative deep molecular response (MR^5.0^) after treatment with single-agent ponatinib. Using CastPCR, we could trace back the presence of the T315I mutation to all the RNA samples up to the detection of T315 mutation by Sanger sequencing shortly after allogeneic hematopoietic stem cell transplantation (HSCT).

**Conclusion:**

This case illustrates the major interest of ponatinib as a valid treatment option for e19a2 CML patients who present a T315I mutation following relapse after HSCT.

**Electronic supplementary material:**

The online version of this article (10.1186/s12885-018-5100-4) contains supplementary material, which is available to authorized users.

## Background

Chronic myeloid leukaemia (CML) is a myeloproliferative neoplasia caused by the fusion of the *BCR* and *ABL1* genes, usually as the result of the reciprocal translocation t(9;22)(q34;q11.2). The exact breakpoint of the translocation and the molecular weight of the resulting fusion gene protein are variable, with most of the breakpoints on chromosome 22 falling in the major breakpoint cluster region (M-BCR), between exons 13 and 14 of the *BCR* gene, leading to a *BCR–ABL1* mRNA with e13a2 or e14a2 junctions encoding for a p210 fusion protein [1]. Less than 5% of patients express atypical transcript types, of which e19a2, with breakpoints in the micro breakpoint cluster region (μ-BCR) and coding for the p230 BCR-ABL1 protein, is the most frequently encountered with a frequency of 0.7–2.7% of the cases [2, 3]. p230 CML has been associated with various clinical presentations and courses with variable responses to first-line imatinib, possibly confounded due to reporting bias in favour of cases with atypical features and/or responses [4–7]. The present study reports the case of a patient with p230 CML that was successfully treated with the third-generation tyrosine kinase inhibitor (TKI) ponatinib, following an early relapse with a T315I mutation after allogeneic stem cell transplantation.

## Case presentation

**Fig. 1:**
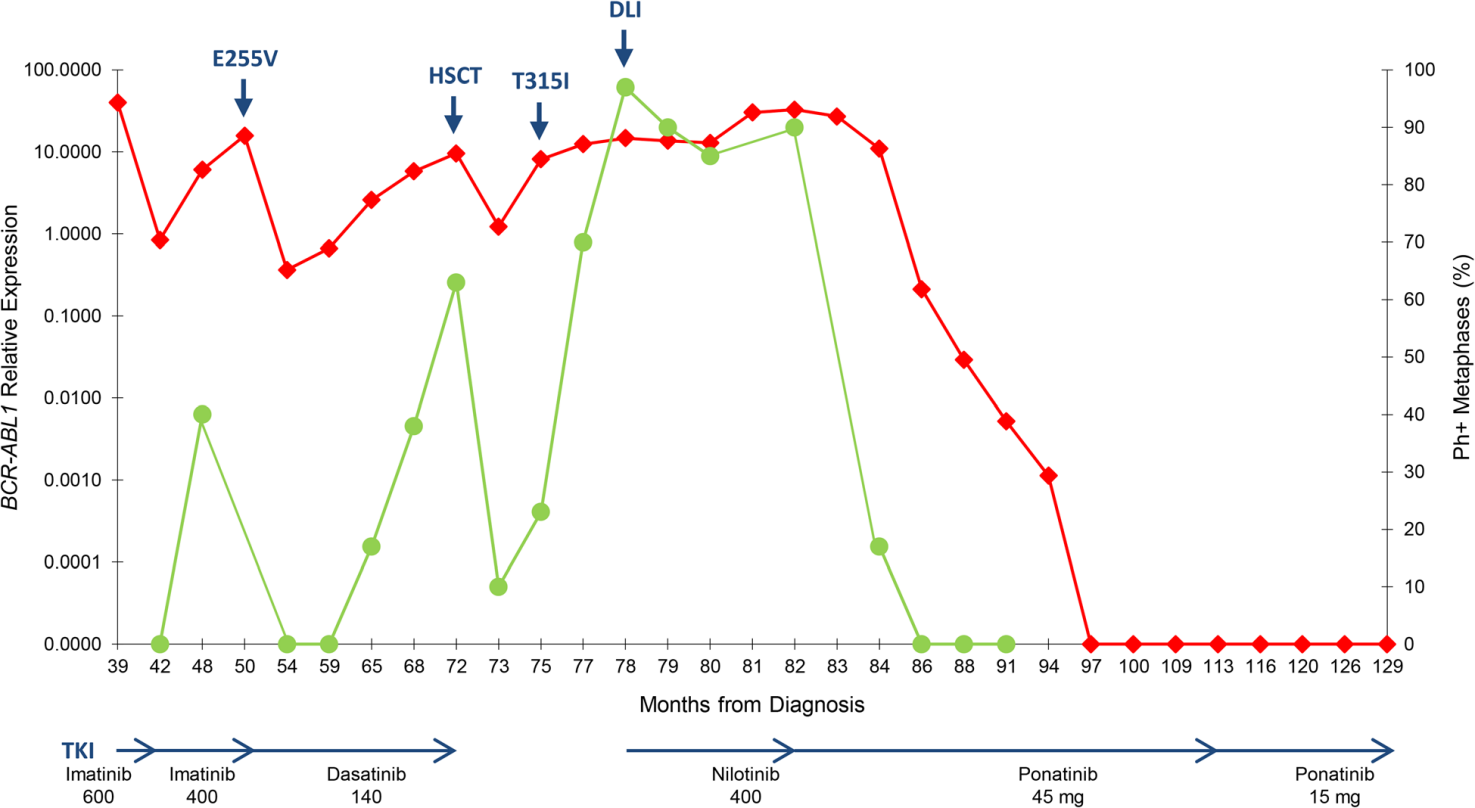
Monitoring of *BCR-ABL1* levels (red) and cytogenetic response (green) during the different treatments of the patient. HSCT, haematopoietic stem cell transplant; DLI, donor lymphocyte infusion

In November 2007, a 51 year-old man was admitted to the Centre Hospitalier Universitaire Vaudois in Switzerland for observation due to leucocytosis. His white blood cell (WBC) count was 232,000/μL, with 130,000/μL neutrophils (69.8%), 2300/μL basophils (1%), 7000/μL eosinophils (3%), 140 × 10^9^/L platelets, 9.5 g/dL hemoglobin, and 2% peripheral blood blasts. He had no liver or spleen enlargement. The calculated Sokal, EUTOS and ELTS scores were low (0.64), low (7), and intermediate (1.639), respectively. A bone marrow aspirate showed marked hypercellularity and myelogenous hyperplasia without an increase in the blast ratio. Cytogenetic analysis showed the presence of a 46,XY,t(9;22)(q34;q11.2),+der(22)t(9;22) karyotype in 25 metaphases, and the presence of a *BCR-ABL1* gene fusion was confirmed by FISH analysis. Molecular analysis revealed the presence of an e19a2 *BCR-ABL1* transcript and the patient was diagnosed with an e19a2-positive chronic phase CML. The detection of an additional Ph+, a major route abnormality that has been reported as an adverse prognostic factor, does not mandate in daily practice a different initial treatment approach [8], therefore imatinib 400 mg QD was started. The patient rapidly achieved an haematological response and, after 3 months of treatment, a partial cytogenetic response (PCyR) was observed (28% Ph + metaphases), corresponding to an optimal response according to the ELN guidelines [8]. After 6 months of imatinib therapy, he lost his cytogenetic response (CyR), with all the metaphases analysed showing the same karyotype detected at diagnosis, which suggested treatment failure [8]. At this point, the patient interrupted the treatment and was lost to follow-up. In May 2010, the patient presented with hyperleukocytosis (300,000/μL), left abdominal pain and malaise. A CT scan revealed a splenomegaly of 20 cm and karyotype analysis showed the presence in 25 metaphases of the same karyotype observed at diagnosis. In view of the patient’s previous history, it was decided to initiate treatment with imatinib 600 mg QD. After 4 months of therapy, he was in haematological remission, but karyotype analysis showed only a minimal cytogenetic response (75% Ph + metaphases). In December 2010, 6 months after starting therapy, his cytogenetic response improved to a minor response (45% Ph + metaphases), corresponding to a sub-optimal response [8]. In February 2011, by decision of the patient, he was transferred to our institute (Additional file 1: Table S1). At the time of admission, he was in complete haematological response. Molecular analysis showed the presence of an e19a2 *BCR-ABL1* transcript, but no cytogenetic analysis was performed. In order to quantitate the e19a2 *BCR-ABL1* transcript and monitor response to treatment, a *BCR* exon 19 forward primer was designed (ENF007; 5′- CACTGAAGGCAGCCTTCGA-3′) and used with reverse primer ENR561 and probe ENP541 (7). A standard curve was established by tenfold dilutions of patient cDNA. Control *ABL1* transcripts were detected as previously described [9]. At this point, 9 months after restarting imatinib therapy, the *BCR-ABL1/ABL1* ratio was 40.258% (Fig. 1). Three months later, at the 12-month evaluation, the patient finally achieved a complete cytogenetic response (CCyR), with a *BCR-ABL1* level of 0.846% (Fig. 1). At this point, and taking into account the good response to treatment, the patient was switched to imatinib 400 QD. However, 6 months later, karyotype analysis showed loss of CCyR (40% Ph + metaphases; minor CyR), with a concomitant rise in *BCR-ABL1* levels (6.070%). Two months later, an additional rise in *BCR-ABL1* levels to 15.820%, confirmed loss of molecular response and prompted search for mutations in the *ABL1* kinase domain as previously reported [10]. Sanger sequencing allowed identification of an E255V mutation (Fig. 1), leading to start of second line dasatinib therapy (140 mg QD), and prompting search for an unrelated stem cell donor. Three months after start of dasatinib therapy, the patient achieved a CCyR with a *BCR-ABL1* level of 0.365% and 5 months later, the patient maintained a CCyR, with a *BCR-ABL1* level of 0.671%. However, in the following months, the patient showed a progressive loss of cytogenetic response: PCyR (17% Ph + metaphases), minor CyR (38% Ph + metaphases), and minor CyR (63% Ph + metaphases), 14 months, 17 months and 21 months after starting second-line dasatinib therapy, respectively. At this time, dasatinib therapy was stopped and the patient underwent allogeneic haematopoietic stem cell transplant (HSCT) from a 9/10 matched HLA unrelated donor (one HLA-B mismatch), after a non-myeloablative reduced-intensity conditioning (RIC) regimen that included busulfan, fludarabine and antithymocyte globulin (ATG). The graft-versus host disease (GVHD) prophylaxis consisted of the association of tacrolimus with mycophenolate mofetil (MMF). Neither acute GVHD nor other significant clinical complications were observed. One month after transplant, cytogenetic analysis showed persistence of disease (10% Ph + metaphases; PCyR), with a *BCR-ABL1* level of 1.229%, which prompted start of nilotinib therapy (400 mg BID). Two months later, his *BCR-ABL1* level rose to 8.132% with 23% Ph + metaphases (PCyR), suggesting resistant disease. Mutational analysis by Sanger sequencing revealed the presence of a T315I mutation, which led to the interruption of both nilotinib therapy and immunosuppression. Four months after transplant the patient lost his PCyR (70% Ph + metaphases; minor CyR) and his *BCR-ABL1* levels increased to 12.477%. The patient showed mixed chimerism predominantly donor-derived. Since ponatinib was not available at the time at our institution and approval was pending, nilotinib therapy (400 mg BID) was restarted. One month later, karyotype analysis showed a minimal CyR (97% Ph + metaphases) and the patient was submitted to donor lymphocyte infusion (DLI), which resulted only in a minor CyR (90% Ph + metaphases) that nevertheless was maintained in the following months. This was, however, accompanied by increasing *BCR-ABL1* levels to a maximum of 32.879%, 10 months after HSCT. The patient also showed progressive loss of donor chimerism (mixed chimerism with < 5% donor cells). At this point ponatinib approval was granted, nilotinib therapy was interrupted and ponatinib was started at the standard dose of 45 mg QD. Before ponatinib initiation, and considering its well-known cardiovascular toxicity profile, a comprehensive cardiologic and metabolic evaluation was performed. The only cardiovascular risk-factors identified were dyslipidaemia and arterial hypertension (AHT), which were already present since the beginning of the disease and were easily manageable with lifestyle modifications and therapy (enalapril/hydrochlorothiazide 20/12.5 mg, ½ tablet QD), respectively. Two months later, karyotype analysis showed a PCyR (17% Ph + metaphases) and a reduction of *BCR-ABL1* levels (10.940%) was observed. Subsequently, four months later, the patient achieved a CCyR associated with a *BCR-ABL1* level of 0.213%. In the following months a progressive decline in *BCR-ABL1* levels was documented: 0.029% at 6 months (equivalent to a Major Molecular Response; MMR), 0.005% at 9 months (equivalent to a MR^4.0^), 0.001% at 12 months (equivalent to a MR^4.5^), and 0.000% at 15 months (equivalent to a MR^5.0^). The patient maintained ponatinib 45 mg QD treatment during an additional 16 months and, since a persistent and sustained MR^5.0^ with no detectable disease was documented, dosage was reduced to 15 mg QD. Treatment with ponatinib was well tolerated since the beginning of therapy with no evidence of toxicity. At the time of the last evaluation, 15 months after ponatinib dose reduction, the patient maintained a sustained MR^5.0^ with no detectable disease. No evidence of cardiovascular toxicity was observed at the last observation. Under ponatinib treatment, the patient maintained a mixed chimerism with < 5% donor cells. To evaluate if the T315I clone was present at low-levels in the earlier phases of the disease, we used CastPCR (Competitive Allele-Specific TaqMan PCR™, Applied Biosystems) specific for the T315I mutation in all the available RNA patient samples collected since he was admitted to our institution. For the T315I mutation detection, each sample was run in real-time PCR with the assay targeting both the mutation and the corresponding reference gene, according to the manufacturer’s instructions. After amplification, data files containing the samples Ct values were imported into Life Technologies Mutation Detector™ Software for post-PCR data analysis. CastPCR is highly specific and sensitive and can detect and quantitate rare amounts of mutated DNA/cDNA in a sample that contains large amounts of normal, i.e., non-mutated, DNA/cDNA, with a sensibility up to 0.1%. Using this approach, we could trace back the presence of the T315I mutation to all the RNA samples up to the detection of T315 mutation by Sanger sequencing shortly after HSCT.

## Discussion and conclusions

p230 CML has been associated with various clinical presentations and courses [4, 5] with recent data suggesting that these patients have a poor response to front-line imatinib therapy, but better responses to second-line nilotinib and dasatinib [6, 7]. Point mutations in the kinase domain (KD) of the *ABL1* gene represent the most common resistance mechanism to TKI therapy in CML, occurring in 30–90% of patients who develop resistance to imatinib [11], with more than 100 different mutations reported, although many are only rarely detected clinically [11]. The relative in vitro sensitivity of different mutants to the various available TKI varies considerably and correlates well with the outcome after subsequent therapy with a different TKI [12]. Similarly, mutants that are less likely to be inhibited by a given TKI are more likely to emerge clinically during therapy with such inhibitors [13].

Here we report a case of imatinib resistance due to an E255V mutation, followed by early post-transplant relapse with a T315I mutation that achieved a persistent negative deep molecular response (MR^5.0^) after treatment with single-agent ponatinib. Recently, patients with E255K/V mutations have been described as having a particularly poor prognosis, regardless of the stage of the disease at detection, with a higher risk of transformation to advanced/blast phase, and a short survival [14]. Indeed, this mutation shows a high IC_50_ to all available TKIs, including third-generation ponatinb [15]. In this case, dasatinib has been suggested as the most likely option in terms of probability of response among all available TKIs [14]. Therefore, dasatinib treatment was initiated as a bridge to HSCT, and a CCyR was quickly achieved. However, in the following months, the patient lost CCyR and at the time of HSCT showed only a minor CyR. The best response achieved after HSCT was a PCyR, that was nevertheless quickly lost with mutation analysis revealing the presence of a T315I. Since the T315 mutation was retrospectively detected even before the detection of the E255V mutation, this suggests that at least two distinct *BCR-ABL1* sub-clones were present at the start of therapy. This observation can be explained by differences in competitive advantage between mutant clones. Indeed, in vitro cell assays showed that selected mutant clones (for example, P-loop mutations Y253F, E255K) have higher transformation potency and proliferation rate compared with T315I, even in the absence of BCR-ABL1 inhibitors [16]. Assuming that imatinib has lower activity against these mutant clones with P-loop mutations, they may expand more rapidly than clones with the T315I mutation when exposed to imatinib [13]. On the other hand, it has been shown that dasatinib suppresses P-loop mutations to a greater extent than T315I [17], therefore a T315I positive clone may be able to increase its size during dasatinib treatment with relatively little competition from rapidly proliferating clones [13]. This hypothesis is supported by the observation that dasatinib treated patients seem to more frequently show T315I mutations [13]. This is in agreement with the model in which evolution of *BCR-ABL1*–positive cells is mainly shaped by TKI-selective pressure and the fitness of each *ABL1* KD mutated population is the net result of the ability to survive treatment depending on the intrinsic sensitivity to the specific TKI administered and of the ability to survive the competition with all other coexisting populations [18].

Although our patient did not develop adverse events related to ponatinib therapy, its expected benefits must be balanced against the potential risks, including arterial hypertension, and serious arterial occlusive and venous thromboembolic events, reported in the PACE trial in 19 and 5% of patients, respectively. Published data support the observation that adverse events appear to be related to certain pre-existing cardiovascular risk factors, ponatinib dose, or both [19]. For this reason, and since our patient was in sustained MR^5.0^ with no detectable disease after 27 months of ponatinib therapy, the dose was reduced to 15 mg OD. Fifteen months later, the patient maintains a sustained MR^5.0^ with no detectable disease. Ponatinib efficacy was previously observed in a CML e19a2 patient refractory to both imatinib and dasatinib therapy due to the presence of a T315I mutation [20]. These results suggest that ponatinib could be an excellent therapeutic option in the treatment of e19a2 CML harbouring the T315I mutation refractory to previous therapies including HSCT, although more studies are necessary to draw a definitive conclusion.

## Additional file

Additional file 1: **Table S1.** Clinical and laboratory data of the patient. (DOCX 17 kb)

## Abbreviations

AHT: Arterial hypertension
ATG: Antithymocyte globulin
CastPCR: Competitive Allele-Specific TaqMan PCR
CCyR: Complete cytogenetic response
CML: Chronic myeloid leukaemia
CyR: Cytogenetic response
DLI: Donor lymphocyte infusion
GVHD: Graft-versus host disease
HSCT: Haematopoietic stem cell transplant
M-BCR: Major breakpoint cluster region
MMF: Mycophenolate mofetil
MMR: Major molecular response
PCyR: Partial cytogenetic response
RIC: Reduced-intensity conditioning
TKI: Tyrosine kinase inhibitor
WBC: White blood cell
μ-BCR: Micro breakpoint cluster region
