# Supplementary material for: Ponatinib induces a sustained deep molecular response in a chronic myeloid leukaemia patient with an early relapse with a T315I mutation following allogeneic hematopoietic stem cell transplantation: a case report
Source: BMC Cancer. 2018 Dec 7;18:1229. doi: 10.1186/s12885-018-5100-4 (PMC6286606; doi:10.1186/s12885-018-5100-4)
Supplement: Supplementary file 1 — Table S1. Clinical and laboratory data of the patient. (DOCX 17 kb) [file 12885_2018_5100_MOESM1_ESM.docx]

**Additional Table 1 – Clinical and laboratory data of the patient**

| Time  (months) | Therapy | Karyotype  Ph+ (%) | CyR | BCR-ABL1% | Molecular Response | BCR-ABL1 Mutation (SS) | T315I (CastPCR) |
| --- | --- | --- | --- | --- | --- | --- | --- |
| 0 | No therapy | 100 | None | NA | NA | ND | NA |
| 3 | Imatinib 400 | 28 | PCyR | NA | NA | ND | NA |
| 30 | Imatinib 600 | 100 | None | NA | NA | ND | NA |
| 34 | Imatinib 600 | 75 | Minimal CyR | NA | NA | ND | NA |
| 36 | Imatinib 600 | 45 | Minor CyR | NA | NA | ND | NA |
| 39 | Imatinib 600 | ND | ND | 40.258 | ≥0.1% | ND | + |
| 42 | Imatinib 600 | 0 | CCyR | 0.846 | ≥0.1% | ND | + |
| 48 | Imatinib 400 | 40 | Minor CyR | 6.070 | ≥0.1% | ND | + |
| 50 | Imatinib 400 | ND | ND | 15.820 | ≥0.1% | E255V | + |
| 54 | Dasatinib 140 | 0 | CCyR | 0.365 | ≥0.1% | ND | + |
| 59 | Dasatinib 140 | 0 | CCyR | 0.670 | ≥0.1% | ND | + |
| 65 | Dasatinib 140 | 17 | PCyR | 2.593 | ≥0.1% | ND | + |
| 68 | Dasatinib 140 | 38 | Minor CyR | 5.795 | ≥0.1% | ND | + |
| 72 | Dasatinib 140 | 63 | Minor CyR | 9.608 | ≥0.1% | ND | + |
| 73 | HSCT | 10 | PCyR | 1.229 | ≥0.1% | ND | + |
| 75 | No therapy | 23 | PCyR | 8.131 | ≥0.1% | T315I | + |
| 77 | No therapy | 70 | Minor CyR | 12.477 | ≥0.1% | ND | ND |
| 78 | Nilotinib 400 + DLI | 97 | Minimal CyR | 14.793 | ≥0.1% | ND | ND |
| 79 | Nilotinib 400 | 90 | Minor CyR | 13.725 | ≥0.1% | ND | ND |
| 80 | Nilotinib 400 | 85 | Minor CyR | 12.963 | ≥0.1% | ND | ND |
| 81 | Nilotinib 400 | ND | ND | 30.382 | ≥0.1% | ND | ND |
| 82 | Nilotinib 400 | 90 | Minor CyR | 32.879 | ≥0.1% | ND | ND |
| 83 | Ponatinib 45 | ND | ND | 27.189 | ≥0.1% | ND | ND |
| 84 | Ponatinib 45 | 17 | PCyR | 10.940 | ≥0.1% | ND | ND |
| 86 | Ponatinib 45 | 0 | CCyR | 0.213 | ≥0.1% | ND | ND |
| 88 | Ponatinib 45 | 0 | CCyR | 0.029 | MMR | ND | ND |
| 91 | Ponatinib 45 | 0 | CCyR | 0.005 | 4.0 | ND | ND |
| 94 | Ponatinib 45 | ND | ND | 0.001 | 4.5 | ND | ND |
| 97 | Ponatinib 45 | ND | ND | 0.000 | 5.0 | ND | ND |
| 100 | Ponatinib 45 | ND | ND | 0.000 | 5.0 | ND | ND |
| 109 | Ponatinib 45 | ND | ND | 0.000 | 5.0 | ND | ND |
| 113 | Ponatinib 15 | ND | ND | 0.000 | 5.0 | ND | ND |
| 116 | Ponatinib 15 | ND | ND | 0.000 | 5.0 | ND | ND |
| 120 | Ponatinib 15 | ND | ND | 0.000 | 5.0 | ND | ND |
| 126 | Ponatinib 15 | ND | ND | 0.000 | 5.0 | ND | ND |
| 129 | Ponatinib 15 | ND | ND | 0.000 | 5.0 | ND | ND |

HSCT - Hematopoietic Stem Cell Transplantation; DLI - Donor Lymphocyte Infusion; CyR, Cytogenetic Response; PCyR, Parcial Cytogenetic Response; CCyR, Complete Cytogenetic response; MMR, Major Molecular Response; MR^4.0^, level 4.0 molecular response; MR^4.5^, level 4.5 molecular response; MR^5.0^ , level 5.0 molecular response; SS; Sanger Sequencing; ND, Not Done; NA, Not Available.
